# Molecular Mechanisms and Therapeutic Potential of Gabapentin with a Focus on Topical Formulations to Treat Ocular Surface Diseases

**DOI:** 10.3390/ph17050623

**Published:** 2024-05-11

**Authors:** Dario Rusciano

**Affiliations:** Fidia Ophthalmic Research, 95123 Catania, Italy; drusciano55@gmail.com

**Keywords:** gabapentin, gamma-amino-butyric-acid (GABA), dry eye, neuropathic pain, eye drops

## Abstract

Gabapentin (GBP) was originally developed as a potential agonist for Gamma-Amino-Butyric-Acid (GABA) receptors, aiming to inhibit the activation of pain-signaling neurons. Contrary to initial expectations, it does not bind to GABA receptors. Instead, it exhibits several distinct pharmacological activities, including: (1) binding to the alpha-2-delta protein subunit of voltage-gated calcium channels in the central nervous system, thereby blocking the excitatory influx of calcium; (2) reducing the expression and phosphorylation of CaMKII via modulation of ERK1/2 phosphorylation; (3) inhibiting glutamate release and interfering with the activation of NMDA receptors; (4) enhancing GABA synthesis; (5) increasing cell-surface expression of δGABA_A receptors, contributing to its antinociceptive, anticonvulsant, and anxiolytic-like effects. Additionally, GBP displays (6) inhibition of NF-kB activation and subsequent production of inflammatory cytokines, and (7) stimulation of the purinergic adenosine A1 receptor, which supports its anti-inflammatory and wound-healing properties. Initially approved for treating seizures and postherpetic neuralgia, GBP is now broadly used for various conditions, including psychiatric disorders, acute and chronic neuropathic pain, and sleep disturbances. Recently, as an eye drop formulation, it has also been explored as a therapeutic option for ocular surface discomfort in conditions such as dry eye, neurotrophic keratitis, corneal ulcers, and neuropathic ocular pain. This review aims to summarize the evidence supporting the molecular effects of GBP, with a special emphasis on its applications in ocular surface diseases.

## 1. Introduction

Gamma-aminobutyric acid (GABA) and glutamate (GLU) play crucial roles in the control of neuropathic pain through their actions within the central nervous system (CNS). These neurotransmitters separately activate two distinct classes of receptors: ionotropic and metabotropic. Ionotropic receptors work as ligand-gated ion channels, inducing rapid synaptic transmission, while metabotropic receptors belong to the G-protein coupled receptor (GPCR) family, contributing to the neuro-modulatory effects mediated by glutamate and GABA. Remarkably, within the pain neuraxis, these receptors can be found at various points along the pathway in the nervous system that processes pain signals, thus regulating how pain signals are transmitted and perceived by the brain [1].

GABA is an amino acid that works as the primary inhibitory neurotransmitter for the CNS. It functions to reduce neuronal excitability by inhibiting nerve transmission. In the context of neuropathic pain, GABAergic inhibitory interneurons modulate the transmission of pain signals within the spinal cord and brain. GABAergic signaling inhibits the activation of pain-signaling neurons, dampening their transmission. GABA exerts its inhibitory effect through two types of specific receptors, GABA_A (ionotropic) and GABA_B (metabotropic), which show different pharmacological, structural, and molecular differences. GABA receptors are the most common in the nervous system. Upon ligand binding, they change their shape slightly to allow negatively charged chloride ions to pass through their channel into the neuron, thus reducing its excitability. Therefore, GABA is classified as an inhibitory neurotransmitter. Dysfunction in GABAergic neurotransmission, such as a decrease in GABAergic inhibitory activity, can lead to hyperexcitability of pain-signaling pathways, contributing to the development and maintenance of neuropathic pain [2].

Glutamate (GLU) is the primary excitatory neurotransmitter in the CNS. Glutamatergic signaling plays a key role in the transmission and amplification of pain signals. In neuropathic pain conditions, excessive release of glutamate and increased activation of glutamate receptors contribute to neuronal hyperexcitability and central sensitization, leading to the amplification and prolongation of pain signals. Glutamate receptors, particularly N-methyl-D-aspartate (NMDA) receptors, are implicated in the development and maintenance of neuropathic pain by mediating synaptic plasticity and the induction of central sensitization [3].

**Figure 1:**
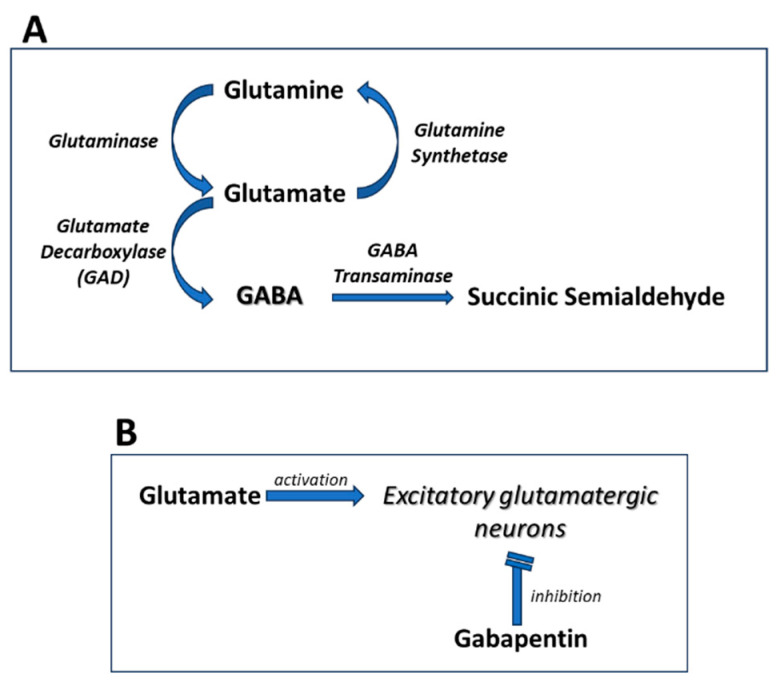
**Mechanisms of Gabapentin Antalgic Action: GABA Synthesis and Glutamatergic Inhibition** (**A**) The pathways leading to GABA synthesis and degradation. (**B**) The analgesic effect of gabapentin depends on the inhibition of excitatory glutamatergic neurons, occurring through mechanisms that do not involve GABA receptors.

Considering the involvement of GABA and glutamate in neuropathic pain, pharmacological interventions aimed at enhancing GABAergic inhibition or modulating glutamatergic excitatory signaling are of significant interest for its management. Medications like gabapentin (GBP), which interact with voltage-gated calcium channels and indirectly influence GABA and glutamate neurotransmitter systems, are commonly employed for their efficacy in alleviating neuropathic pain (Figure 1).

## 2. Pharmacology of GBP Action

GBP is a non-anesthetic drug with analgesic and anticonvulsant properties. It has demonstrated efficacy in the treatment of partial epileptic seizures and neuropathic pain [4,5]. GBP was made by the addition of a cyclohexyl group to GABA, which allowed this form of GABA to cross the blood–brain barrier [6]. 

**Figure 2:**
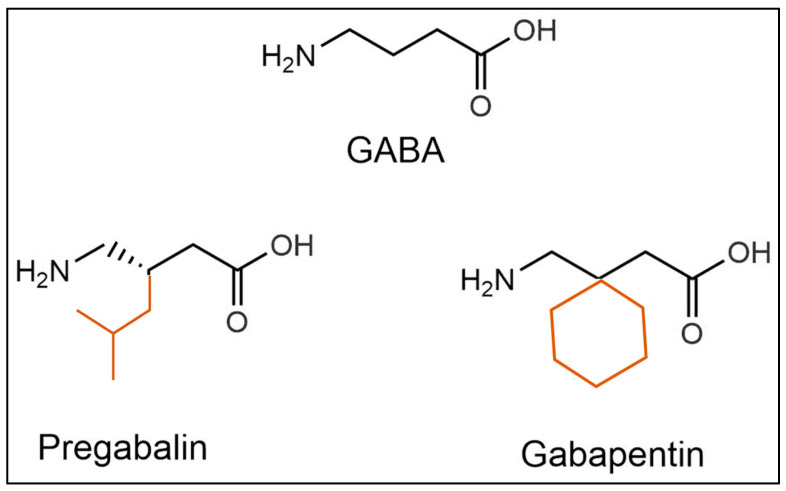
**Gabapentinoids in Pharmacology**: Structurally related to gamma-aminobutyric acid (GABA), Gabapentin does not appear to interact with GABA receptors or influence its synaptic reuptake [8]. Pregabalin is an alternative pharmaceutical form of gabapentin, showing very similar properties [9]. Gabapentin and pregabalin are collectively indicated as gabapentinoids. The chemical additions to GABA to obtain pregabalin or gabapentin are colored orange-red.

GBP was originally developed to mimic the neurotransmitter GABA to potentially act as an agonist at GABA receptors, so as to inhibit the activation of pain-signaling neurons (Figure 2). However, it does not significantly bind to GABA_A or GABA_B receptors and does not directly influence GABA uptake or degradation [7]. The mechanisms through which GBP exerts its effects are somewhat different from the initial hypothesis and include the following.

### 2.1. Effects on Voltage-Gated Calcium Channels

**Figure 3:**
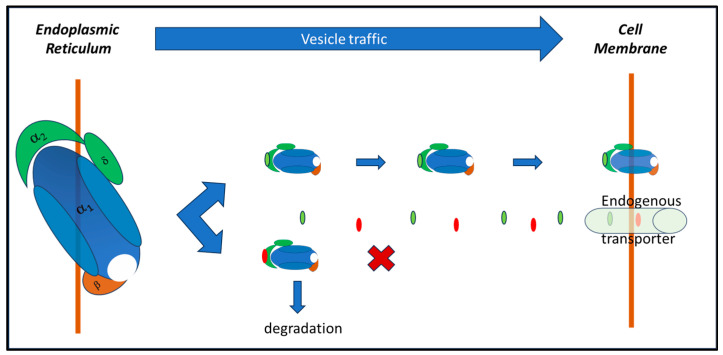
**Hypothetical mechanism of GBP effects on CaVα2-δ channels.** GBP (red dots) takes advantage of the endogenous L-leucine (light-green dots) transporter to enter the cell, and competitively binds the α2 subunit of the voltage-gated calcium channel, blocking its transport to the cell membrane, thus reducing calcium influx into the cell, and its activation.

At the neuronal level, GBP inhibits the activity of voltage-dependent L-type calcium channels by binding to the α2-δ1 and α2-δ2 subunits. It also acts as an inhibitor on voltage-dependent N-type calcium channels. In this way, it reduces the influx of calcium ions into presynaptic terminals and decreases the release of excitatory neurotransmitters, such as glutamate. This action contributes to its anticonvulsant and analgesic effects [8,10]. These studies describe the effects of GBP on calcium channel currents and trafficking, shedding light on its mechanism of action. The binding of GBP to the α2-δ1 and α2-δ2 subunits of voltage-gated calcium channels has little acute effect on calcium currents. At the same time, chronic exposure to GBP inhibits calcium currents in heterologous expression systems and dorsal root ganglion neurons. This inhibition occurs when GBP is included for 17–20 h before recording, but not if included for 3–6 h. This suggests that GBP’s inhibition of calcium currents requires chronic exposure. GBP primarily acts at an intracellular location, requiring uptake into cells. This effect is mediated by α2-δ subunits of calcium channels, as mutations preventing GBP binding abolish its inhibitory effect [10]. GBP disrupts the trafficking of α2-δ2 and calcium channel subunits, reducing their cell-surface expression. This disruption occurs chronically and is not observed with acute GBP exposure. These findings suggest that GBP may displace an endogenous ligand (L-leucine) that positively modulates α2-δ subunit function, leading to impaired trafficking of calcium channels [8]. GBP’s effects on calcium channel currents and trafficking contribute to its mechanism of action (Figure 3), potentially explaining its therapeutic effects in conditions such as epilepsy and neuropathic pain.

### 2.2. Effects on CaMKII

Experimental evidence has suggested that Ca^2+^/calmodulin-dependent protein kinase II (CaMKII) may contribute to GBP’s analgesic effects in a rat model of chronic constriction injury (CCI). GBP or saline were given to rats undergoing CCI, and mechanical allodynia and thermal hyperalgesia were assessed. GBP significantly reduced pain sensitivity in treated rats, with maximal effects observed on postoperative day 8. Additionally, GBP decreased the expression and phosphorylation of CaMKII in the spinal cord of CCI rats. These findings suggest that GBP’s analgesic effects in treated rats may be mediated, at least in part, by modulation of CaMKII expression and phosphorylation in the spinal cord [11].

### 2.3. Effects on Glutamate Receptors

GBP may reduce excitatory neurotransmission by decreasing GLU release, thereby interfering with the activation of NMDA (N-methyl-D-aspartate) receptors. This mechanism is likely to contribute to its anticonvulsant properties and potentially also its analgesic properties. A study in a rat neuropathic pain model demonstrated that systemic or intrathecal GBP administration effectively reduced mechanical and cold hypersensitivity, with intrathecal administration showing superior efficacy. GBP also attenuated the formalin-induced release of GLU and aspartate in the spinal cord dorsal horn, suggesting its ability to alleviate neuropathic pain symptoms by inhibiting glutamate release in this region [12]. In a different model system, GBP effects were evaluated on neurotransmitter-gated ion channels and GIRK (G protein-coupled inwardly rectifying potassium) channels in the spinal cord, hypothesizing it as a possible site of action. Xenopus laevis oocytes genetically manipulated to express human receptors/channels revealed no effect of GBP on GABA(A) receptors, glycine receptors, or GIRK channels, even at high concentrations. However, GBP concentration-dependently inhibited NMDA receptors, with significant inhibition at 10 microM. Notably, at 30 microM, GBP competitively inhibited NMDA receptors without altering their response curve, suggesting a non-competitive inhibition. Glycine attenuated this effect in a concentration-dependent manner. These findings indicated that GBP’s antinociceptive property may be attributed to its inhibition of NMDA receptors, rather than GABA(A) receptors, glycine receptors, or GIRK channels [13]. Additionally, pre-treatment with GBP protected rat hippocampal CA1 neurons against NMDA-induced excitotoxicity by inhibiting NMDA receptor-activated ion currents, thus attenuating glutamate-induced neuronal injury [14], finally confirming the inhibitory effects of GBP on the GLU/NMDA-receptor system. Even more interesting is the recent finding that the α2-δ1 subunit of voltage-gated ion channels, a known binding site for gabapentinoids used to manage neuropathic pain and epilepsy, plays a significant role in neuropathic pain hypersensitivity through its association with NMDA receptors. Specifically, overexpression of Cacna2d1, the gene encoding α2-δ1, increased both presynaptic and post-synaptic NMDA receptor activity in spinal dorsal horn neurons, leading to pain hypersensitivity. Conversely, knockdown or removal of Cacna2d1 normalized the increased NMDA receptor activity induced by nerve injury. Further investigation revealed that α2-δ1 forms a complex with NMDA receptors in both rodent and human spinal cords, predominantly through its C terminus, promoting the surface trafficking and synaptic targeting of NMDA receptors. GBP or a peptide interfering with the C terminus of α2-δ1 normalized NMDA receptor synaptic targeting and activity increased by nerve injury. These findings highlight α2-δ1 as a critical NMDA receptor-interacting protein that enhances its synaptic delivery in neuropathic pain. Moreover, Gabapentinoids alleviate neuropathic pain by inhibiting the forward trafficking of α2-δ1-NMDA receptor complexes [15].

### 2.4. Effects on GABA Synthesis

Although GBP does not act directly on GABA receptors, it appears to enhance the GABA synthetic pathway. In fact, in laboratory settings, GBP enhances the function of glutamic acid decarboxylase (GAD), responsible for synthesizing GABA, and inhibits GABA-transaminase, responsible for its degradation, but only at high concentrations. Studies using nuclear magnetic resonance (NMR) spectroscopy on human and rat brains suggested that GBP enhances the production of GABA [16]. Therefore, by increasing the synthesis of GABA, GBP may indirectly enhance inhibitory neurotransmission, although this effect does not appear to be the primary mechanism of action. However, further studies did not confirm these findings, rather reporting that GBP boosted the presence of δGABA_A receptors and heightened a steady inhibitory current in neurons. This rise in expression probably plays a role in the calming effects of GABA, as evidenced by GBP-induced reductions in coordination and anxiety in normal mice, but not in mice lacking the δ subunit. Interestingly, both normal and δ subunit-lacking mice experienced pain relief from GBP, indicating the presence of other different mechanisms of action, independent of GABA receptors. Notably, levels of GABA_A receptor activators and neuro-steroids in the brain remained unchanged in response to GBP [17]. Accordingly, a most recent study reported that gabapentin and pregabalin did not acutely increase cellular GABA levels in the healthy rat forebrain. Instead, they modestly decrease cellular glutamate levels [18]. These findings further support the hypothesis that, while gabapentin and pregabalin may modulate glutamate levels, their primary mechanism of action may not involve direct modulation of GABA levels in the healthy rat brain.

### 2.5. Anti-Inflammatory Effects

GBP also exerts an anti-inflammatory action, as demonstrated in different model systems. In vitro, using human neuroblastoma cells (SH-SY5Y) and rat glioma cells (C6) [19] demonstrated that GBP can counteract the activation of the NF-kB factor induced by Substance P (SP), involved in the induction of inflammatory episodes. In vivo, in a rat model of neuropathic pain, GBP increased the levels of IL-10, an anti-inflammatory cytokine capable of inhibiting interferon-gamma (INFγ), and the expression of pro-inflammatory cytokines, like TNF-α, IL-1, IL-6, IL-8, and IL-12 [20]. The anti-inflammatory activity of GBP has also been demonstrated in a murine model of carrageenan-induced peritonitis. GBP treatment reduced the production of inflammation mediators and pro-inflammatory cytokines, neutrophil infiltration, and oxidative stress [21]. Most recently, the anti-inflammatory effects of GBP on ventricular remodeling post-myocardial infarction (MI) were investigated in a rat model of MI, and by in vitro experiments with the THP-1 macrophage cell line. In vivo, GBP administration following MI led to reduced expression of the pro-inflammatory marker inducible nitric oxide synthase (iNOS) and decreased levels of inflammatory cytokines, such as TNF-α and IL1β. Additionally, GBP promoted the expression of markers associated with anti-inflammatory M2 macrophages and increased levels of anti-inflammatory factors, such as chitinase-like 3, IL-10, and TGFβ. Furthermore, GBP treatment improved cardiac function, reduced infarct size, and decreased cardiac fibrosis. In vitro experiments demonstrated that GBP reduced the expression of pro-inflammatory factors in LPS-stimulated macrophages. This effect was partially mediated by PPAR-γ activation and involved downregulation of α2δ1 calcium channel expression in macrophage membranes, leading to decreased intracellular calcium levels. Overall, GBP mitigated cardiac remodeling post-MI by suppressing inflammation through PPAR-γ activation and calcium regulation [22].

### 2.6. Effects on Adenosine A1 Receptors 

GBP has also been reported to interact with other molecular targets, such as the stimulation of the adenosine A1 receptor subtype, involved in modulating neurotransmitter release, neuronal excitability, and inflammation. Adenosine A1 receptors were supposed to play a role in the anticonvulsive effects of certain drugs, including GBP, in a mouse model of seizures induced by the mitochondrial toxin 3-nitropropionic acid (3-NPA) [23]. This study investigated the impact of adenosine receptor antagonists on the anticonvulsive effects of various drugs, including diazepam, phenobarbital, valproate, and GBP. The results showed that antagonists of adenosine A1 receptors, such as aminophylline and 8-cyclopentyl-1,3-dipropylxanthine (DPCPX), diminished the protective effects of these drugs against 3-NPA-induced seizures. This suggests that the activation of adenosine A1 receptors may contribute to the anticonvulsive potential of these medications in this particular model. Moreover, the study found that the non-penetrating adenosine A1/A2 receptor antagonist, 8-(p-sulfophenyl)theophylline (8pSPT), was ineffective, indicating that the central action of adenosine A1 receptors is crucial for the observed effects. Furthermore, the antagonists aminophylline and DPCPX reversed the protective effects of adenosine A1 receptor agonists, suggesting that adenosine A1 receptor stimulation is involved in the anticonvulsant actions of these agonists. Overall, these results imply that the activation of central adenosine A1 receptors may contribute to the anticonvulsive effects of diazepam, phenobarbital, valproate, and GBP in the context of 3-NPA-induced seizures [23]. Another study used a rat model system of mechanical allodynia induced by nerve ligation injury. In this case, GBP and an adenosine A1 receptor agonist, R-PIA, were administered intrathecally to investigate their individual and combined effects on the neuropathic pain symptoms. The obtained results indicated that both GBP and R-PIA produced dose-dependent reductions in mechanical allodynia when administered individually. Moreover, when GBP and R-PIA were co-administered, they synergistically enhanced the antiallodynic effect. This synergistic interaction suggests that GBP potentiates the efficacy of R-PIA in alleviating mechanical allodynia. Additionally, intrathecal administration of an adenosine A1 receptor antagonist reversed the maximal antiallodynic effect observed in the combination group, indicating that activation by GBP and R-PIA of adenosine A1 receptors at the spinal level is necessary for their synergistic interaction on mechanical allodynia [24]. A further study used animal models of complex regional pain syndrome type-I and partial sciatic nerve ligation (PSNL), in which the efficacy of GBP in alleviating neuropathic pain-like behavior was investigated. Neuropathic pain was induced by unilateral prolonged hind paw ischemia and reperfusion (I/R) or PSNL procedures, resulting in stimulus-evoked mechanical hyperalgesia. GBP administration over 3 weeks led to a dose-dependent inhibition of mechanical hyperalgesia in the animals, thus suggesting that GBP effectively alleviated neuropathic pain-like behavior in these animal models. Furthermore, the study explored the contribution of adenosine receptor subtypes to the anti-hyperalgesic effect of GBP. Administration of caffeine, a non-selective adenosine A1 and A2 receptor antagonist, or 1,3-dipropyl-8-cyclopentylxanthine (DPCPX), a selective adenosine A1 subtype receptor antagonist, alongside GBP blocked its anti-hyperalgesic effect. This indicates that the mechanism underlying GBP’s efficacy may involve the activation of adenosine A1 subtype receptors [25]. Overall, these findings suggest that GBP effectively reduces neuropathic pain-like behavior in animal models, and this effect may be mediated, at least in part, by the activation of adenosine A1 subtype receptors.

**Figure 4:**
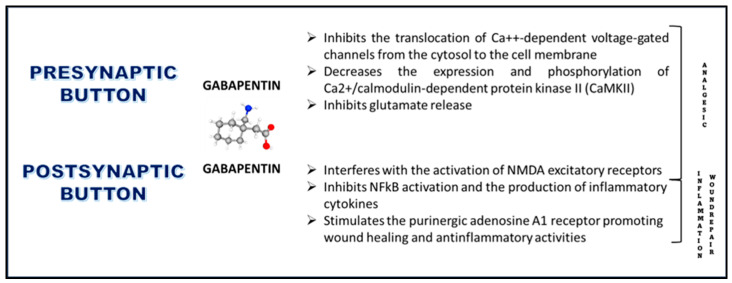
**Pharmacological activities of gabapentin.** The interactions of gabapentin occur at both pre- and post-synaptic terminals of neurons and involve various mechanisms leading to analgesic, anti-inflammatory, and wound-healing effects.

In conclusion, gabapentinoids, including GBP, have emerged as valuable adjuncts in perioperative pain management, aiming to minimize nociceptive input and mitigate the risk of central sensitization [26]. Despite their classification as calcium channel blockers, the precise mechanisms underlying their analgesic effects remain incompletely understood. While their efficacy in neuropathic pain is well-established, their role in postoperative pain management is less defined. Research into the mechanisms of action and effects of gabapentinoids, particularly in experimental animal models and human studies, provides insights into their analgesic properties. Gabapentinoids exert their analgesic effects through multifaceted mechanisms that collectively modulate pain perception. At the spinal level, gabapentinoids inhibit calcium-mediated neurotransmitter release by targeting α2δ-1 subunits, thereby reducing the transmission of pain signals in the dorsal horn. Additionally, they depress dorsal horn sensitivity by interfering with the forward trafficking and recycling of α2δ-1 subunits from dorsal root ganglion neurons. This modulation of dorsal horn sensitivity contributes to the attenuation of pain signaling. Furthermore, gabapentinoids stimulate the uptake of glutamate by excitatory amino acid transporters, potentially dampening excitatory signals in pain pathways. They also inhibit descending serotonergic facilitation, thereby preventing the amplification of pain signals and reducing the risk of central sensitization. Conversely, gabapentinoids promote descending inhibition, augmenting the body’s endogenous pain-suppression mechanisms. Beyond their actions at the spinal level, gabapentinoids exert anti-inflammatory effects, which can mitigate inflammation-induced pain. Moreover, they may influence the affective component of pain, contributing to overall pain relief. While animal studies consistently demonstrate the effectiveness of gabapentinoids in inflammatory and postoperative pain models, clinical evidence in human models is variable. This suggests the need for further research to elucidate the clinical utility of gabapentinoids in postoperative pain management and optimize their therapeutic use. In summary, the analgesic effects of gabapentinoids in perioperative pain management stem from their multifaceted actions on pain pathways, encompassing modulation of neurotransmitter release, dorsal horn sensitivity, descending pain modulation, anti-inflammatory actions, and affective pain processing. Understanding these mechanisms is crucial for optimizing their clinical use and improving pain management outcomes in surgical patients (Figure 4).

### 2.7. Effects on Wound-Healing

Finally, GBP also facilitated wound healing as shown in two different experimental contexts, both involving rat models, which highlighted varied outcomes depending on the formulation and application of GBP. In the first study, the effects of GBP and pregabalin on wound healing in non-diabetic rat models were evaluated [27]. Here, both GBP and pregabalin appeared to delay wound healing initially compared to a control group treated with saline, with increased inflammation noted up to day 13. However, between days 13 and 21, GBP exhibited better healing outcomes than pregabalin. In a second study [28], GBP was conjugated with melittin (a peptide found in bee venom) and formulated into nanoparticles to treat diabetic wounds in rats. This nanoconjugate showed enhanced wound healing properties, evidenced by expedited wound contraction, significant antioxidant activities (preventing malondialdehyde accumulation and promoting activity of superoxide dismutase and glutathione peroxidase), and superior anti-inflammatory effects (inhibiting the expression of pro-inflammatory cytokines, like IL-6 and TNF-α). Additionally, it supported the proliferation phase of healing by increasing the expression of growth factors, such as TGF-β, VEGF-A, and PDGFR-β, and boosted the synthesis of hydroxyproline and collagen (Col 1A1). This suggests that GBP, when used in a specific nanoconjugate formulation, can significantly enhance various phases of the wound healing process in diabetic models, including inflammation modulation, oxidative stress reduction, and tissue regeneration. From these studies, it can be inferred that GBP has a complex role in wound healing that may vary significantly based on the model of injury (e.g., diabetic vs. non-diabetic wounds), the stage of wound healing, and the specific formulation and combination with other compounds. GBP’s efficacy seems to improve when it is part of a targeted delivery system, such as in the form of a nanoconjugate, which can enhance its bioavailability and therapeutic impact on wound healing processes (Figure 4).

## 3. Current Therapeutic Applications of Systemic GBP

### 3.1. Psychiatric Disorders

GBP is primarily known for its use in treating neurological conditions, such as epilepsy and neuropathic pain. Its role in the treatment of psychiatric disorders is less established and generally considered off-label [29]. Nonetheless, some evidence suggests that GBP may have potential therapeutic benefits in certain psychiatric conditions. While it appears to have some benefits for anxiety disorders, GBP has shown limited benefit in bipolar disorder and has been used in alcohol craving, withdrawal symptoms, and as an adjunctive therapy for opioid dependence [30]. A recent review indicates that GBP demonstrates efficacy in treating alcohol withdrawal and alcohol use disorder. It is supported as a third-line treatment option for social anxiety disorder and severe panic disorder, backed by sufficient evidence. However, the evidence does not support its use in conditions such as bipolar disorder, major depressive disorder (MDD), posttraumatic stress disorder (PTSD), obsessive-compulsive disorder (OCD), stimulant use disorder, or opioid withdrawal [31].

### 3.2. Anxiety Disorders

Some studies have explored the use of GBP in the treatment of anxiety disorders, including generalized anxiety disorder (GAD), social anxiety disorder (SAD), and panic disorder. GBP has been investigated as an adjunctive treatment to traditional anxiolytics or as a monotherapy. While findings are mixed, some studies suggest that GBP may have anxiolytic effects and could be beneficial in reducing anxiety symptoms [32,33]. This action of GBP appears to work through its binding to the α2δ-1 subunit of voltage-gated calcium channels. This subunit is also involved in binding with thrombospondin, a glycoprotein that facilitates cell–matrix interactions and can modulate synaptic formation and the function of neural circuits. These interactions influence processes such as nociception (pain perception) and potentially other neurological functions [34]. However, research has shown that individuals with a previous history of substance use disorders are at an increased risk of abusing Gabapentinoids, a class of drugs that includes GBP and pregabalin. When these medications are misused alongside illegal drugs, the harmful effects associated with drug abuse can become more severe. Evidence of rising misuse of Gabapentinoids has been confirmed by drug screening practices and analyses performed during autopsies, which have noted higher levels of these substances. Due to the growing concerns about their potential for abuse, many countries have taken regulatory measures by categorizing Gabapentinoids as controlled substances. This classification aims to curb abuse by imposing stricter regulations on prescription, distribution, and consumption [35].

### 3.3. Substance Use Disorders

Emerging evidence, albeit conflicting, suggests that GBP may offer benefits in the treatment of substance use disorders, notably alcohol use disorder and opioid withdrawal. GBP has been proposed to alleviate cravings and withdrawal symptoms, potentially enhancing treatment outcomes for individuals struggling with substance use disorders [36,37].

### 3.4. Bipolar Disorder

GBP’s potential utility in bipolar disorder primarily concerns its mood-stabilizing and anxiolytic effects. It has been explored as a treatment option for managing various symptoms of bipolar disorder, including anxiety, agitation, and mood swings. Some studies suggest that GBP can be beneficial for patients who experience rapid cycling or mixed states in bipolar disorder. The evidence from controlled clinical trials regarding GBP’s efficacy in bipolar disorder remains inconclusive. Several small studies and clinical reports have suggested that it may help manage symptoms of bipolar disorder, particularly in patients who are resistant to other treatments. However, larger and more rigorous studies have generally not confirmed these findings. As a result, GBP is not typically considered a first-line treatment or a standard therapy for bipolar disorder by current clinical guidelines [38,39]. GBP’s mechanism involves binding to the α2δ subunit of voltage-gated calcium channels, which might modulate neurotransmitter release and neuronal excitability—factors that could potentially affect mood regulation. Despite this, the exact mechanism by which GBP might exert mood-stabilizing effects remains unclear. In clinical practice, GBP is sometimes used off-label when other mood stabilizers are ineffective, not tolerated, or contraindicated [40]. Its relatively benign side effect profile compared to other mood stabilizers can also make it a preferred option for certain patients. While GBP has some potential in managing certain aspects of bipolar disorder, especially in complex, treatment-resistant cases or as an adjunct treatment, it should not be used as a primary treatment for bipolar disorder without careful consideration and monitoring by a healthcare provider. Further research is needed to better define its role and efficacy in bipolar disorder management.

### 3.5. Insomnia

GBP has been explored as a potential treatment for pathologic insomnia, particularly in individuals with comorbid psychiatric conditions such as anxiety, restless leg syndrome (RLS), or bipolar disorder. Some studies have found that GBP may improve sleep quality and reduce insomnia symptoms, possibly by modulating neurotransmitter systems involved in sleep regulation [39,41,42]. Contrasting evidence stemmed from other reports [43].

Overall, while GBP’s role in the treatment of psychiatric disorders is not as well-established as its use in neurological conditions, there is some evidence suggesting potential benefits in certain conditions. However, more research is needed to clarify its efficacy, safety, and optimal dosing strategies in psychiatric settings. Additionally, GBP should be used cautiously and under close supervision, as it can have side effects and may interact with other medications used to treat psychiatric disorders.

### 3.6. Pain Management

GBP was originally developed as an analgesic and, indeed GBP’s efficacy for neuropathic pain and postherpetic neuralgia is well-established, with its use as monotherapy or adjunctive therapy supported by case reports and reviews [44]. GBP treatments have also shown efficacy in diabetic peripheral neuropathy [45]; postherpetic neuralgia (PHN) [46], trigeminal neuralgia [47]; neuropathic pain further to paclitaxel chemotherapy [48]; and cancer-related pain in pregnant patients [49]. Pain control in pediatric patients has also been described [50,51].

## 4. Experimental Evidence of Ophthalmic Use of GBP

### 4.1. Inflammation and Neuropathic Ocular Pain

The impact of GBP on the inflammatory response was evaluated in rabbit corneal cells (SIRC) stimulated with lipopolysaccharide (LPS). The study investigated the expression of various inflammatory markers, including TNF-α, IL-1β, cPLA2, COX-2, and PGE2, in corneal cells treated with or without GBP following LPS stimulation. GBP treatment notably decreased the production of cytokines, activation of cPLA2, expression of COX-2, and levels of PGE2 in SIRC corneal cells [52]. A seminal study [53] investigated the response to GBP treatment in patients affected by dry eye accompanied by features of neuropathic ocular pain (NOP). Their findings suggested that GBP treatment may be effective, particularly in patients with systemic comorbidities and less pain response evoked by mechanical and chemical stimuli, indicating its potential for refractory cases not fully responsive to conventional topical treatments. A similar study, published one year later [54], evaluated the efficacy of GBP treatment in dry eye disease and neuropathic ocular pain, demonstrating its effectiveness in improving ocular surface discomfort when combined with artificial tear and cyclosporine drops treatments. Oral GBP also demonstrated analgesic efficacy in clinical studies on postoperative pain of patients subjected to photorefractive keratectomy [55,56,57]. The role of GBP in pain control was also explored through its use in managing postoperative pain following corneal collagen crosslinking (CXL) procedures for keratoconus. Patients were either treated with GBP or ketorolac post-surgery, and their pain levels were assessed using a numeric pain scale. The findings indicate that GBP, administered in 300 mg capsules every 8 h for the first 3 days after surgery, was as effective as ketorolac in controlling pain. There were no significant differences in pain scores between the two groups at any assessment point, nor were there notable differences in eye symptoms or systemic side effects related to the medications. This suggests that GBP is a viable option for pain management after corneal collagen crosslinking procedures, comparable to ketorolac in efficacy [58]. Moreover, a case report indicated GBP as an efficient analgesic intervention to control pain in a painful, blind glaucomatous eye [59]. Additionally, a case report by [60] highlighted the successful resolution of chronic neuropathic ocular pain with GBP, emphasizing its utility in addressing ocular discomfort even in the absence of significant ophthalmic findings. Aside from pain control, a GBP derivative has shown interesting neuroprotective activities. GBP-lactam (GBP-L), a derivative of GBP, exhibited significant neuroprotective effects on retinal ganglion cells (RGCs) across several experimental models of ischemia and neurodegeneration. The primary mechanism underlying these protective effects involves the modulation of mitochondrial ATP-sensitive potassium (K_ATP) channels, which play a crucial role in cellular survival during metabolic stress [61]. GBP-L’s ability to diminish glutamate release under ischemic-like conditions has been particularly notable. Glutamate, a neurotransmitter that becomes a potent neurotoxin under stress conditions, can induce significant neuronal damage and death via excitotoxic pathways. By reducing the ischemia-induced glutamate release, GBP-L minimizes these excitotoxic insults to neurons, thereby enhancing their survival. In vivo studies further underscore the efficacy of GBP-L in enhancing the survival of retinal ganglion cells following acute retinal ischemia. Importantly, GBP-L’s protective effects are evident not only when administered before the onset of ischemia but also when given during the reperfusion phase. This suggests its potential application in clinical settings, such as in the treatment of optic neuropathies and glaucoma, where mitigating ischemic damage is crucial [62]. Interestingly, GBP, the parent compound of GBP-L, does not exhibit similar neuroprotective properties in these models, indicating a distinct pharmacokinetic and pharmacodynamic profile for GBP-L. The uncharged nature of GBP-L possibly allows it to penetrate cellular membranes more effectively than GBP, facilitating its access to intracellular sites of action, particularly within mitochondria. The neuroprotective mechanism of GBP-L has been further elucidated through pharmacological experiments using glibenclamide, a blocker of K_ATP channels. The reversal of GBP-L’s neuroprotective effects by glibenclamide highlights the role of these channels in mediating its actions. Moreover, selective mitochondrial K_ATP channel blockers, such as 5-hydroxydecanoate, have also been shown to negate the survival-promoting effects of GBP-L, confirming the specificity of its action on mitochondrial components of the K_ATP channels [63]. In conclusion, the research on GBP-L presents it as a potent neuroprotective agent with specific actions on mitochondrial K_ATP channels, offering a promising therapeutic approach for conditions characterized by ischemic and excitotoxic neuronal damage. This distinct mechanism and enhanced efficacy compared to its precursor, GBP, positions GBP-L as a potential novel treatment for neurodegenerative diseases affecting the retina and other neuronal tissues.

These studies collectively underscore the promising role of GBP as a potential systemic therapeutic intervention for managing ocular surface discomfort, particularly in cases with neuropathic components that may be refractory to conventional treatments.

### 4.2. Topical Formulation and Treatment with GBP Eye Drops

**Figure 5:**
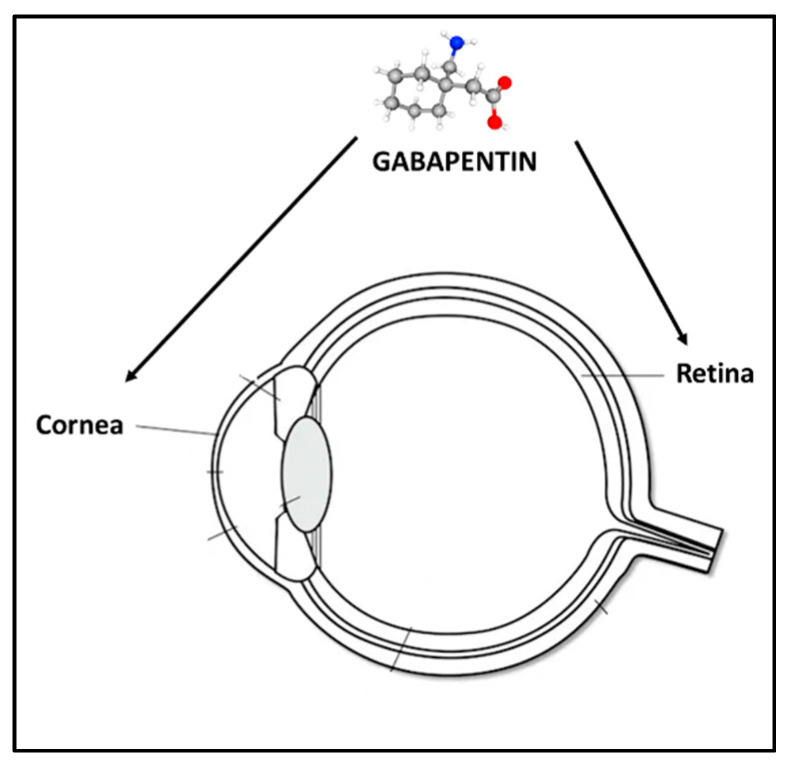
**Ophthalmic application of gabapentin.** Gabapentin has demonstrated efficacy in managing ocular pain associated with dry eye and/or corneal ulcers. Additionally, its secretagogue activity aids in preserving ocular surface hydration. Its neuroprotective properties may contribute to the survival of retinal ganglion cells in conditions such as glaucoma or retinal dystrophies.

Considering the properties of systemic GBP mentioned earlier, topical administration as eye drops could preserve its therapeutic benefits for the eye while minimizing systemic side effects associated with systemic delivery. An ophthalmic formulation of 0.5% GBP was prepared in an isotonic buffered solution (pH 7.0; 298 mOsm). Endotoxin-induced uveitis (EIU) was induced in rabbits by injecting LPS into the eye, and clinical signs of ocular inflammation were evaluated in tears, aqueous, cornea, conjunctiva, and iris-ciliary body at 7 and 24 h post-injection with or without topical application of 0.5% GBP. GBP significantly reduced both clinical symptoms and inflammation biomarkers compared to the LPS group at both time points [52]. These findings suggest that an ophthalmic formulation containing GBP could be beneficial in treating inflammatory conditions associated with ocular pain, like uveitis, and further clinical research is warranted to validate this potential application. Moreover, its main mechanism of action makes GBP an analgesic with no anesthetic effects. GBP primarily acts by modulating the activity of voltage-gated calcium channels in the central nervous system. By inhibiting calcium influx, GBP reduces the release of excitatory neurotransmitters such as glutamate and substance P, which are involved in pain signaling pathways. This modulation of neurotransmitter release results in a reduction of pain transmission, making GBP an effective analgesic agent. This is relevant for topical applications on the eye because an anesthetic would be expected to decrease lacrimation, with negative effects on eye lubrication and corneal pain. In a recent study on rabbit eyes, it was indeed shown that topical application of GBP as eye drops (formulated at 2% in PBS) exhibited analgesic properties without inducing anesthesia, in comparison to a typical anesthetic drug such as oxybuprocaine [64]. Interestingly, GBP not only failed to reduce tear production but unexpectedly increased it. This increase seems to be mediated by the upregulation of acetylcholine and norepinephrine, along with the induction of aquaporin 5 (AQP5) expression in the lacrimal gland. Furthermore, in vitro experiments on the primary human corneal epithelial cell line HCE-F [65] suggested that GBP directly stimulates AQP5 expression in corneal cells. This implies that corneal cells may contribute to the enhanced tear production stimulated by GBP, working alongside the lacrimal glands to restore the tear film and reduce friction on the ocular surface, a known trigger for ocular pain. These results were further corroborated by a recent publication, in which a ceria-based nanocarrier system encapsulating GBP was designed to effectively alleviate dry eye symptoms [66]. Nanoceria eye drops are a type of ocular medication that utilizes nanotechnology to deliver cerium oxide nanoparticles (nanoceria) to the eye. These nanoparticles, typically ranging in size from a few nanometers to a few hundred nanometers, have garnered attention for their potential therapeutic properties and applications in treating various ocular conditions. Nanoceria particles are generally considered biocompatible, meaning they are well-tolerated by living organisms and are unlikely to cause significant adverse reactions when applied topically to the eye [67]. One of the most notable characteristics of nanoceria is their antioxidant activity. Nanoceria particles possess the ability to scavenge reactive oxygen species (ROS) and neutralize oxidative stress, which can be beneficial for protecting ocular tissues from damage caused by oxidative stress-related conditions, such as dry eye disease, age-related macular degeneration (AMD), and diabetic retinopathy [68]. Nanoceria nanoparticles have also been shown to exhibit anti-inflammatory properties, which can help alleviate inflammation and discomfort associated with various ocular conditions, including dry eye disease and uveitis [67,68]. Nanoceria eye drops can serve as a platform for delivering therapeutic agents, such as drugs or bioactive molecules, to the eye. By encapsulating or attaching drugs to the surface of nanoceria particles, it may be possible to enhance drug stability, improve ocular bioavailability, and prolong drug release, leading to improved therapeutic outcomes. Therefore, a multifunctional nanoceria coated with thiolated gelatin and cross-linked with glutaraldehyde was developed [66], thus creating a nanocarrier that is biocompatible and exhibits antioxidant, anti-inflammatory, antiangiogenic, antiapoptotic, and neuroprotective properties. The abundant thiol groups on the gelatin significantly increased the cellular uptake of the nanocarrier by 2.3 times and enhanced its ability to bind mucin by 10 times, improving retention in the eye and boosting the efficacy of the treatment. The moderate cross-linking of the gelatin not only increased the bioavailability of the nanoceria in the eye but also allowed for a controlled, slow release of GBT, which aids in stimulating tear production and restoring the tear film. In a rabbit model of DE, topical application of this GBT/nanoceria nano-formulation provided comprehensive relief from symptoms, repairing corneal epithelial damage, preserving nerve density in the cornea, and enhancing tear secretion, thereby outperforming the free drug. These findings highlight the potential and safety of this novel nano-formulation for advancing DE pharmacotherapy (Figure 5).

Finally, taking advantage of the pleiotropic properties of GBP, including its documented role in promoting wound repair [27,28], while acknowledging one isolated finding in which wound healing was initially delayed by systemic GBP [27], preliminary evidence suggested that the application of sterile GBP solutions directly into the eyes of patients with corneal ulcers could enhance corneal lesion healing, without notable adverse effects. Indeed, anecdotal data from a patent application (WO 2017129577 A1) highlighted the beneficial effects of topical GBP eye drops (0.5% GBP with 0.15% hyaluronic acid) on corneal ulcer healing across a range of patients, including three children aged 2, 3, and 4 years, and two adults aged 77 and 85 years, encompassing both sexes.

## 5. Final Considerations

Final considerations can be made concerning the comparison between gabapentinoids (GBP and pregabalin) in their clinical use to treat neuropathic pain. Some studies show that gabapentin can be beneficial in managing neuropathic pain, with similar efficacy to pregabalin in the case of spinal cord injury [69,70] and chronic neuropathic pain [71]. In the case of post-herpetic neuralgia, pregabalin might work better for controlling PHN pain, but GBP may cause fewer side effects [72]. Similarly, GBP and pregabalin had similar analgesic effects in the treatment of ocular pain, after photorefractive keratectomy (PRK) [57] or chronic neuropathic ocular pain [73]. A review of published studies [74] explored medications used to manage pain after PRK eye surgery. Researchers analyzed 23 studies and found that most medications (including GBP and pregabalin) offered similar pain relief. Tetracaine, an anesthetic, and nepafenac, an NSAID, appeared most effective for pain control. However, both topical anesthetics and NSAIDs had a potential side effect of slowing down corneal healing, with tetracaine causing the most significant delay. The study suggests that combining topical NSAIDs and anesthetics, particularly tetracaine, might need to be avoided to minimize the risk of compromising corneal healing. Instead, we know that GBP, also given topically, has a good analgesic effect, does not decrease lacrimation [64], and does not impair wound healing [28]. 

## 6. Conclusions

In conclusion, gabapentin (GBP) shows promise as a topical treatment for ocular surface conditions due to its analgesic, anti-inflammatory, and secretagogue properties. This could prove particularly beneficial for managing neuropathic pain and dry eye disease. Eye drop formulations offer the advantage of localized delivery, potentially minimizing systemic side effects compared to oral administration. While both gabapentin and pregabalin share structural similarities with leucine and may utilize similar transporters, further research is necessary to determine definitively which is superior for topical ocular applications. Existing studies involving topical GBP eye drops merit further investigation. I personally believe that the way has been paved to start the path leading to the registration and production of an innovative topical treatment for ocular surface diseases, with the added benefit of analgesic effects, in such cases where pain is a complication of the pathology. 

## Data Availability

Data sharing is not applicable.
